# INTERGENERATIONAL REMINISCENCE AND DIGITAL STORYTELLING: EXPLORING THE EXPERIENCE OF COLLEGE STUDENT VOLUNTEERS

**DOI:** 10.1093/geroni/igad104.1089

**Published:** 2023-12-21

**Authors:** Noelle Fields, Ling Xu, Brooke Troutman, Kathryn Daniel, Allyson Miles

**Affiliations:** The University of Texas at Arlington, Arlington, Texas, United States; The University of Texas at Arlington, Arlington, Texas, United States; Air Force Academy, Air Force Academy, Colorado, United States; University of Texas at Arlington, Arlington, Texas, United States; University of Texas at Arlington, Arlington, Texas, United States

## Abstract

Research suggests that reminiscence combined with an intergenerational approach may yield significant psychosocial benefits for older adults with Alzheimer’s disease and related dementia (ADRD). Through intergenerational reminiscence, older persons may pass on their life experience and life lessons to younger generations. Intergenerational contact and relationships between older and younger adults can prevent stereotypes or negative attitudes from forming, or counter existing ones. Additionally, outcomes from intergenerational programming include the younger generation’s ability to build relationships with older adults and have positive changes in attitudes toward aging. The current paper is part of a larger telephone-based intervention that used intergenerational reminiscence with digital storytelling in sample of university student volunteers and community-dwelling older adults with ADRD. University student volunteers were provided with training before the intervention about the myths and facts about ADRD, communicating with persons with ADRD, and how to create a digital story. Using Rapid and Rigorous Qualitative Data Analysis (RADar), a research team examined the weekly written reflections of the student volunteers (N = 27) during the 10-week pilot study. The analysis yielded 6 themes: positive connections, resilience, life lessons, empathy, collaboration, and growth. Many students reported increased confidence in talking with older adults and shared an interested in remaining connected after the study ended. Few students reported technological challenges with the DST. Findings from the study extend the existing literature related to volunteer-based interventions for older adults with ADRD and highlight the potential of involving younger adults in new approaches to ADRD research and practice.

